# Offense related characteristics and psychosexual development of juvenile sex offenders

**DOI:** 10.1186/1753-2000-3-19

**Published:** 2009-07-11

**Authors:** Lisette 't A Hart-Kerkhoffs, Theo AH Doreleijers, Lucres MC Jansen, Anton PH van Wijk, Ruud AR Bullens

**Affiliations:** 1 VU University Medical Center, Department of Child and Adolescent Psychiatry, Amsterdam, the Netherlands; 2 Beke Research and Consultancy, Arnhem, the Netherlands; 3 VU University, Department of Forensic Child and Juvenile Psychology, Amsterdam, the Netherlands

## Abstract

**Objective:**

This article reports on offense related characteristics and the psychosexual development in subgroups of juvenile sex offenders as measured by the Global Assessment Instrument for Juvenile Sex Offenders (GAIJSO). The predictive validity of these characteristics for persistent (sexual) offensive behavior in subgroups of juvenile sex offenders was investigated.

**Methods:**

One hundred seventy four sex offenders (mean age 14.9 SD 1.4) referred by the police to the Dutch Child Protection Board were examined. Offense related characteristics were assessed by means of the GAIJSO and the BARO (a global assessment tool for juvenile delinquents), and criminal careers of the subjects were ascertained from official judicial records.

**Results:**

Serious need for comprehensive diagnostics were found on the domains sexual offense and psychosexual development in juvenile sex offenders, especially in the group of child molesters. These youngsters displayed more internalizing and (psychosexual) developmental problems and their sexual offense was more alarming as compared to the other juvenile sex offender subgroups. Although one third of the juveniles had already committed one or more sex offenses prior to the index offense, at follow up (mean follow up period: 36 months SD 18 months) almost no sexual recidivism was found (0.6% of the entire sample). However, a substantial proportion of the entire sample of juvenile sex offenders showed non-sexual (55.6%) and violent recidivism (32.1%). Several predictors for a history of multiple sex offending and non-sexual recidivism were identified.

**Conclusion:**

This study revealed numerous problems in juvenile sex offenders. Assessment using the GAIJSO is helpful in order to identify indicators for extensive diagnostic assessment. In order to investigate the predictive validity for sexual reoffending a longer follow up period is necessary.

## Background

It has been estimated that about 20% of all rapes and 20–50% of cases of child abuse are perpetrated by juveniles [1]. Identifying those youngsters who are at risk for persistent sexual deviant and offensive behavior will avert more victimization at an early stage. However, in offending minors, despite the volumes of research on the characteristics of these juveniles, distinguishing transient incidental sexual misdemeanor from more persistent offensive (sexual) behavior may be difficult, because literature on this specific topic is scarce.

This article reports on sex offense related characteristics and the psychosexual development of subgroups of juvenile sex offenders. The predictive validity of these characteristics for persistent (sexual) offensive behavior, in subgroups of juvenile sex offenders, was investigated.

Sexual offending by juveniles is frequently considered to be experimental behavior as a part of normal sexual development. However, previous studies have shown that many adolescent sex offenders had prior victims [2,3], and indicated that their behavior is similar to that of adult paraphilic sex offenders. Some juvenile sex offenders do seem to have paraphilic sexual impulses and behavior patterns [4] and some adolescent sex offenders seem to have underlying deviant sexual arousal patterns or urges [5], similar to that of adult sex offenders. Therefore, in the assessment of juvenile sex offenders it seems important to distinguish normal from deviant sexual development, in order to know which of these boys is at risk for repetitive sexual offending. On the one hand, this is necessary for better protection of potential future victims; on the other hand, because public policy is becoming increasingly punitive. To date, even many low risk adolescents are subjected to long term specific treatment programs and public registration, which may also have long term negative effects on the development of these youngsters [6].

The literature on juvenile sexual recidivism is scarce, and the identification of predictors of sexual reoffending is hampered by the relatively small sample sizes of these studies, differences in length of follow up and differences in the definition of sexual recidivism. In 22 published follow up studies of juvenile sex offenders, sexual assault recidivism rates ranged from 0–40% and general (including sexual) recidivism was found in 11–89% [7]. However, it seems that juvenile sex offenders are generally more likely to reoffend non-sexually than sexually [8,9]. Variables that may predict future offending among juvenile sex offenders (e.g. offender age, criminal history and antisocial personality) also predict reoffending among juvenile non-sex offenders [10,8,11], and many of these variables reflect general antisocial tendencies [10].

As to specific risk factors for sexual reoffending in juveniles, many risk factors are mentioned in the literature, but few have been empirically supported. Deviant sexual interest [12,13] seems to be a strong predictor of sexual reoffense, especially in child molesters [14]. Some studies elicited socially isolated juvenile sex offenders [15,13], who displayed poor social skills turned out to reoffend sexually more often than those without such difficulties. Victim related characteristics such as not being a relative or friend [16,17], having assaulted more than one victim [18], as well as deviant sexual interests, prior criminal sanctions for sexual assault(s) and drop out from offense specific treatment [19] have been reported to be sexual reoffense predictors.

The BARO (Basis Raads Onderzoek: Protection Board Preliminary Investigation of Criminal Cases) [20]; is a global assessment tool for juvenile delinquents (12–18 year olds) designed for the Child Protection Board (CPB) in order to standardize and improve the quality of CPB assessments. As the BARO itself does not provide information on specific risk factors for deviant sexual development and/or recidivism in juvenile sex offenders, a specific assessment paragraph for juvenile sex offenders was developed, to be used in addition to the BARO: the Global Assessment Instrument for Juvenile Sex Offenders (GAIJSO) [Dutch original version: Screeningsinstrument voor Jeugdige Zedendelinquenten (SIJZ); [21,22]. The GAIJSO collects information on offense related characteristics and risk factors for persistent (sexual) offensive behavior. It leads to insight into the characteristics of the sexual offense and to an estimation of the severity of the offense, as well as evaluating the psychosexual development of the offender. The BARO and GAIJSO together yield data to advise the justice authorities and indicates subsequent treatment. The GAIJSO has an advantage over other measures such as MASA, which was originally developed for adult sex offenders [23], because it includes self report data as well as data from police files. Moreover, GAIJSO can easily be used in addition to the BARO, a global assessment tool for juvenile delinquents (12–18 year olds), which is used in several countries.

This article reports on offense related characteristics and psychosexual development, as measured by the GAIJSO, and the predictive validity of these characteristics for persistent (sexual) offensive behavior in subgroups of juvenile sex offenders.

## Methods

### Setting and subjects

As the police in the Netherlands are obliged to refer all 12 to 18 year olds suspected of having committed a (sex) crime to the CPB, a number of regional CPB offices were the primary site of inclusion. Four (out of 22) regional CPB offices were selected for participation based on their location in both rural and urban regions in the Netherlands. Part of this group was admitted to a juvenile justice institution (JJI) subsequent to their arrest. Male juvenile suspects of sex offenses admitted to four (out of 13) JJIs, to which the selected CPB regional offices usually referred, were asked to participate as well. Exclusion criteria were an IQ below 70 and insufficient command of the Dutch language. The study was approved by the Ethics Committee of the VU University Medical Center Amsterdam, The Netherlands. After explaining the study to the subjects, informed consent was obtained from them and their parents or legal guardians.

It should be noted that although participants were legally considered to be only *suspected *of committing a sexual offense (i.e. arrested/charged for a sexual offense), for the sake of readability in this article they will be called sex offenders or sexual delinquents.

### Measurements

#### File information

Offense characteristics such as age and gender of the victim and number of (co)offenders were retrieved from both police records and CPB files. Based on characteristics of the index offense, juvenile sex offenders were divided into three subgroups:

1) child molesters: offenders who had sexually abused children (below 12 years of age) who were four or more years younger than the offender himself (n = 30).

2) solo peer offenders: offenders who had raped or sexually assaulted peers (at least twelve years old) or older persons on their own (n = 54).

3) group offenders: offenders who had raped or sexually assaulted peers (at least twelve years old) or older persons in a group, consisting of (at least) two or more offenders (n = 90).

#### Global assessment of risk and protective factors (BARO) [20]

Psychometric properties of the BARO were described as good, with its discriminative validity being good to excellent for predicting the presence of psychopathology [20,24]. The BARO has been translated into German, English, Russian and Finnish. The validation study of the German version showed comparable properties. The BARO covers nine domains of problems and functioning: delinquent behavior, environmental conditions, psychosocial development, externalizing problems, internalizing problems, substance use, functioning within the family and school and leisure-time functioning. For each of the domains, a number of individual questions are administered to the youth and his parent. At the end of the interview, each of the nine domains of the BARO can be rated with respect to the degree of concern: no, some, high and very high concern (or: no information). Based on domain scoring, an estimation of the seriousness of the youth's problems is made and advice to the justice authorities is formulated. For the purpose of this study, domain scores were dichotomized into two levels of concern: 1) no or some concern versus 2) high or very high concern. The unknown variables were considered missing variables.

#### Global Assessment Instrument for Juvenile Sex Offenders (GAIJSO) [21]

The GAIJSO is developed to use in clinical practice, in order to estimate the severity of the sexual offense and to make an evaluation of the psychosexual development. The most important risk factors for sexual recidivism and deviant psychosexual development found in the literature have been included in the GAIJSO. The items were formulated by a group of Dutch clinical and scientific experts in the field of juvenile sex offenders. Altogether, this instrument consists of 25 items divided into two scales: the offense scale (15 items) and the psychosexual development scale (10 items). The sexual offense-items are scored with information derived from the juvenile sex offender and from police files. The psychosexual development-scale contains information derived from the youngster. Each item contains a group of semi-structured questions and has several response options (example of items: empathy: shows no, some, adequate empathy for the victim; violence: no violence (including verbal aggression), used 'enough' violence (including verbal aggression) to take control over the victim, used excessive violence to take control over the victim (for example: used a weapon or physically injured the victim). Deviant sexual development is defined as a development towards behavior in which sexual gratification is obtained through unusual practices that are harmful or humiliating to others (or self), criminal or socially repugnant. Deviant sexual fantasies are defined as sexual fantasies that can be part of a deviant sexual development and that can lead to criminal conduct in real life. At the end of each scale a global assessment is given of the level of concern regarding the sexual offense itself and the psychosexual development. The GAIJSO leads to a qualitative estimation of the necessity of requesting extensive diagnostic assessment. In order to adjust to the jargon of the CPB counselors, the word 'concern' has been chosen.

An interview with the perpetrator as to these items in addition to the BARO, completed with information derived from police files, results in an estimation of the severity of the sexual offense and an evaluation of the youth's psychosexual development. This information can contribute to a well considered advice to the justice authorities and identifies indicators for extensive diagnostic assessment. The two domains of concern of the GAIJSO have six concern options: no, some, high or very high (or: unknown/unclear). For the analyses we clustered these options into no or some concern (not problematic or non-case) and high or very high concern (problematic or case). For these analyses we considered the unknown and unclear variables to be missing variables. All items of the GAIJSO are shown in table 1. The GAIJSO-interviews were done by a total of fifteen CPB counselors, who received training by the authors.

**Table 1:** Items GAIJSO

I. Sexual offense	II. Psychosexual development
1. Victim age	16. Sex education
2. Gender of victim	17. Sexual preoccupation
3. Relation victim and perpetrator	18. Deviant sexual fantasies
4. Nature of sexual act	19. Sexual excitement during offense
5. Type of offending (solo versus as part of a group)	20. Deviant sexual behavior
6. Place in group	21. Previous sex offenses
7. Offense history (frequency)	22. History of sexual victimization
8. Offense history (duration)	23. Sexualizing family
9. Confession to/denial of the offense	24. Deviant sexual attitudes/perversities
10. Taking responsibility	25. Global assessment care domain psychosexual development
11. Empathy	
12. Insight into risk situations and 'triggers'	
13. Planning/preparation	
14. Use of force	
15. Global assessment care domain sexual offense	

#### Recidivism

At follow up (T1, mean follow up period 36 months SD 18 months), the rate of (sexual) recidivism was determined through official registration systems from the Dutch police (HKS: *Herkenningsdienstsysteem*, Police Identification Service System) and the Dutch Ministry of Justice (JDS: *Justitieel Documentatie Systeem*, Judicial Documentation System). From these registration systems, all (past) criminal activities (arrests, charges) were extracted for each juvenile sex offender in this study. Both sexual and non-sexual offenses were listed and of the non-sexual offenses violent offenses were listed separately. By means of these official registration systems, recidivism of the offenders as well as their criminal history prior to the index offense could be investigated.

### Statistical analyses

The data were processed and analyzed using SPSS (Statistical Package for the Social Sciences, version 13.0). An extensive description of the population of juvenile sex offenders was done with descriptive statistics. Differences between subgroups were analyzed using Chi-square tests (or Fisher exact test if necessary) in the case of categorical variables. ANOVAs (Analysis of variance) or student's t-tests were used in the case of continuous variables. Finally, logistic regression analyses (forward conditional method) were performed for predicting non-recidivism versus recidivism and single versus multiple sex offending. Variables with a p-value < 0.2 in the bivariate analysis were included as predictors.

## Results

In total, 309 boys were eligible for the study, of whom 226 (73%) agreed to participate (mean age 14.98, SD 1.39). Non-responders did not differ from responders with respect to age (t = .232; (300); p = .817), or offense characteristics such as type of offending (χ^2 ^= .094 (2); p = .954), gender of the victim (χ^2 ^= .782 (2) p = .676) or age of the victim (χ^2 ^= .130 (1); p = .719). However, responders were more often of non-Dutch ethnicity than non-responders (59% versus 44%, χ^2 ^= 4.198; p < 0.05). Complete GAIJSO data were available for 77% of the responders (n = 174). Participants had a mean age of 14.9 years (SD: 1.4) and almost forty percent of these boys were of Dutch origin. Child molesters were significantly more often of Dutch origin as compared to solo and group offenders.

Results for the nine BARO domains for juvenile sex offender(s) (subgroups) are shown in table 2. Based on the offense domain scores, extensive diagnostic assessment was deemed necessary in more than seventy percent of the juvenile sex offenders.

**Table 2:** BARO domains

	**Total group** N = 174		**Solo offenders** N = 54		**Group offenders** N = 90		**Child molesters** N = 30		
*High/very high concern*	*n*	***%***	*n*	***%***	*n*	***%***	*n*	***%***	*χ^2^; (2, N = 174); p*

BARO offense	106	**71.6**	26	**60.5**_a_	56	**71.8**_a, b_	24	**88.9**_b_	6.595; p < 0.05
BARO environment	65	**43.9**	16	**37.2**	34	**43.6**	15	**55.6**	2.274; .321
BARO development	54	**37.0**	16	**38.1**_a_	21	**27.3**_a_	17	**63.0**_b_	10.957; p < 0.05
BARO externalizing	67	**45.6**	20	**46.5**	33	**42.9**	14	**51.9**	.673;.714
BARO internalizing	44	**31.2**	12	**29.3**_a_	17	**23.0**_a_	15	**57.7**_b_	10.905; p < 0.05
BARO substance abuse	9	**6.4**	2	**4.9**	4	**5.5**	3	**11.5**	1.402; .496
BARO family	40	**26.8**	15	**34.9**_b_	12	**15.4**_a_	13	**46.4**_b_	12.099; p < 0.05
BARO school	42	**28.6**	13	**31.0**	20	**25.6**	9	**33.3**	.745; .689
BARO leisure time	59	**39.9**	13	**30.2**	34	**43.0**	12	**46.2**	2.425; .297

*Note*. Percents with same subscripts do not differ significantly at p < 0.05 by post-hoc 2 × 2 chi-square analysis.

BARO: Global assessment of risk and protective factors

Significant differences between subgroups were found on the following domains: offense, development, internalizing problems. These differences were mostly accounted for by child molesters. As compared to solo offenders, more concern was indicated for child molesters on the domain offense. Child molesters had more problems as compared to both other subgroups on the domains development and internalizing problems. Problematic familial circumstances were more frequently detected among child molesters as compared to group offenders.

Results of the GAIJSO are shown in table 3 and 4.

**Table 3:** GAIJSO sexual offense characteristics

	**Total group** N = 174		**Solo offenders** N = 54		**Group offenders** N = 90		**Child molesters** N = 30		
	*N*	*%*	*N*	*%*	*N*	*%*	*N*	*%*	*χ^2^; (2, N = 174); p*

Victim age > 4 yrs younger	30	**17.2**	0	**0**_a_	0	**0**_a_	30	**100**_b_	174.000; p < 0.05
Male victim	24	**13.8**	7	**13.0**_a_	3	**3.3**_a_	14	**46.7**_b_	35.578; p < 0.05
Unknown victim	42	**24.3**	19	**35.8**_b_	21	**23.3**_b_	2	**6.7**_a_	8.965; p < 0.05
Penetration	95	**54.6**	18	**33.3**_a_	58	**64.4**_b_	19	**63.3**_b_	14.294; p < 0.05
Group offending (leader)	95	**54.6**	0	**0**_a_	90 (20)	**100_c_(25)**	5	**16.7**_b_	157.191; p < 0.05
≥2 sex offenses	60	**34.5**	24	**44.4**	22	**24.4**	14	**46.7**	8.357; .015
≥2 sex offenses: < 6 months	30	**57.7**	10	**43.5**_a_	16	**94.1**_b_	4	**33.3**_a_	14.062: p < 0.05
Denial	92	**54.4**	25	**49.0**_a_	58	**65.9**_b_	9	**30.0**_a_	12.496; p < 0.05
Taking no responsibility	60	**35.1**	21	**38.9**	33	**37.9**	6	**20.0**	3.650: .161
Lack of empathy	75	**44.1**	25	**47.2**	39	**44.8**	11	**36.7**	.894; .640
Insight into risks and 'triggers'	28	**18.5**	12	**25.5**	14	**18.2**	2	**7.4**	3.743; .154
Impulsive offense	63	**38.0**	16	**29.6**	38	**44.7**	9	**33.3**	3.479; .176
Use of force	87	**54.0**	21	**42.0**_a_	53	**64.6**_b_	13	**44.8**_a, b_	7.615; p < 0.05
Excessive violence	18	**11.2**	8	**16.0**_b_	10	**12.2**_b_	0	**0**_a_	4.905; p < 0.05

**Global Assessment** High/very high concern	103	**59.5**	26	**49.1**_a_	21	**56.7**_a_	26	**86.7**_b_	11.890; p < 0.05

*Note*. Percents with same subscripts do not differ significantly at p < 0.05 by post-hoc 2 × 2 chi-square analysis.

GAIJSO: Global Assessment Instrument for Juvenile Sex Offenders

**Table 4:** GAIJSO psychosexual development problems

	**Total group** N = 174		**Solo offenders** N = 54		**Group offenders** N = 90		**Child molesters** N = 30		
	*N*	***%***	*N*	*%*	*N*	*%*	*N*	*%*	*χ^2^; (2, N = 174); p*

Insufficient sex education	106	**60.9**	34	**63.0**	55	**61.1**	17	**56.7**	.324; .850
Sexual preoccupation	100	**59.5**	29	**54.7**	49	**57.0**	22	**75.9**	3.953; .139
Deviant sexual fantasies	26	**15.7**	1	**2.0**_a_	16	**18.8**_b_	9	**30.0**_b_	12.560; p < 0.05
Sexual excitement	33	**20.5**	8	**15.1**_a_	14	**17.9**_a_	11	**36.7**_b_	6.074; p < 0.05
Deviant sexual behavior	38	**22.5**	12	**23.1**	18	**20.7**	8	**26.7**	.472; .790
Previous sex offenses	37	**21.3**	17	**31.5**	15	**16.7**	5	**16.7**	4.882; .087
Sexual victimization	20	**11.5**	7	**13.0**_a, b_	6	**6.7**_a_	7	**23.3**_b_	6.310; p < 0.05
Sexualizing family	13	**7.5**	3	**5.6**	6	**6.7**	4	**13.3**	1.836; .399
Deviant attitudes/perversities	72	**42.1**	18	**34.0**_a_	45	**51.1**_b_	9	**30.0**_a_	6.189; p < 0.05

**Global Assessment** High/very high concern	65	**38.2**	17	**32.7**_a_	29	**33.0**_a_	19	**63.3**_b_	9.718; p < 0.05

*Note*. Percents with same subscripts do not differ significantly at p < 0.05 by post-hoc 2 × 2 chi-square analysis.

GAIJSO: Global Assessment Instrument for Juvenile Sex Offenders

### Sexual offense (see table 3)

In almost one fifth of the sexual offenses the victim was a child. Approximately 55% of the offenses were perpetrated in a group. A quarter of the peer/adult group offenders were initiators or leaders of the offense. Of the total group, in about 14% the victim was male and in almost one quarter the victim was not known to the perpetrator. Forty five percent of the perpetrators admitted to have penetrated their victims. One quarter of the offenders was suspected of having committed more than one sexual offense and in almost 60% these offenses took place within a period of six months. Although about half of the subjects initially denied their participation in the offense, later in the interview, almost two thirds of the offenders did admit some responsibility. Almost 45% of the offenders did not show any empathy for the victim. Less than 20% of the perpetrators reported adequate insight into risk situations and 'triggers'. In about 40% the offenses were considered to be impulsive acts, because there seemed to be no preparation preceding the offense. Violence was used in more than half of the offenses, of which one fifth was excessive violence. According to the global assessment of the sexual offense, concern was indicated in almost 60% of the offenders (see table 3).

### Psychosexual development (see table 4)

More than 60% of the boys received insufficient sex education. Sexual preoccupation was reported in almost 60% of the offenders and approximately 16% reported to have deviant sexual fantasies. Almost a quarter of the offenders admitted to exhibiting deviant sexual behavior, but only one fifth admitted to feeling sexual excitement during this deviant behavior. More than one fifth of the perpetrators said they had previously committed sexual offenses that were not reported to the police. A history of sexual victimization of themselves occurred in almost 12% of the total group. More than 40% of the offenders displayed deviant sexual attitudes. Concern was indicated, on the global assessment of the psychosexual development, in about 40% of the offenders.

Differences between subgroups of juvenile sex offenders (see tables 3 and 4):

More child molesters compared to solo offenders and group offenders were rated as high concern on the global assessment domains indicating a more alarming sexual offense and more precarious psychosexual development. As compared to the other two subgroups, child molesters more often had abused a male, acquainted victim, more frequently admitted to feeling sexual excitement, and showed no excessive violence at all. As to their psychosexual development, more child molesters had a history of being victimized sexually compared to group offenders. Fewer solo offenders penetrated their victims as compared to child molesters and group offenders. Moreover solo offenders showed fewer deviant sexual fantasies compared to child molesters and group offenders. Compared to both other subgroups, group offenders denied the offense more frequently, showed more deviant attitudes and in cases of multiple sex offenses they offended more frequently in a shorter period of time. More group offenders than solo offenders used violence during the offense.

### Criminal career (see table 5)

**Table 5:** Criminal career

	**Total group** N = 162		**Solo peer offenders** N = 49		**Group offenders** N = 84		**Child molesters** N = 29		
	*N*	***%***	*N*	*%*	*N*	*%*	*N*	*%*	*χ^2 ^(2, N = 162); p*
**Prior to index**									

Sexual offense	52	**32.1**	19	**38.8**	23	**27.4**	10	**34.5**	1.936; .380
Non-sex offense	53	**32.7**	11	**22.4**_a_	35	**41.7**_b_	7	**24.1**_a, b_	6.373; .041
Violent offense	34	**21.0**	7	**14.3**_a, b_	24	**28.6**_b_	3	**10.3**_a_	6.221; .045

**Recidivism**									

Sexual offense	1	**0.6**	0	**0**	1	**1.2**	0	**0**	0.934; .627
Non-sex offense	90	**55.6**	23	**46.9**_a_	58	**69.0**_b_	9	**31.0**_a_	14.728; .001
Violent offense	52	**32.1**	12	**24.5**_a_	35	**41.7**_b_	5	**17.2**_a_	7.767; .021

									F; (df); p

TaR (months) (= mean follow up period)	36 (SD 18)		33 (SD 20)		38 (SD 15)		38 (SD 22)		1.145;(2, 159); .321

*Note*. Percents with same subscripts do not differ significantly at p < 0.05 by post-hoc 2 × 2 chi-square analysis.

Results regarding the criminal careers of juvenile sex offenders are given in table 5.

Group offenders displayed more non-sexual and violent offensive behavior before the index offense and at follow up. Although a substantial proportion of the subjects committed more than one sexual offense before or at the time of the index offense, hardly any (0.6%) sexual recidivism was found at follow up. However, a substantial number (56%) of the juvenile sex offenders showed non-sexual and violent recidivism.

GAIJSO variables that were shown to be associated with non-sexual and violent recidivism in chi-square analyses (p < 0.2) were included in a logistic regression analysis (see table 6). Because sexual recidivism occurred in only one subject at follow up, this variable could not be included in the analysis. Non-sexual recidivism was predicted by group offending, taking no responsibility and insufficient sex education and was negatively associated with having a male victim. Furthermore, violent (non-sexual) reoffending was predicted by group offending, insufficient sex education and lack of empathy.

**Table 6:** Predictive value of various risk factors for non-sexual, violent and multiple sex offending

	***B***	***SE***	***β(95% CI)***
**Non-sexual recidivism** *Full model: chi-square 27.338, df 4, N = 140, Nagelkerke R^2 ^0.237*			

Group offending	0.998	.386	2.982 (1.494–5.952)*
Male victim	-1.083	.558	0.338 (0.113–1.010)*
Taking no responsibility	1.160	.471	3.190 (1.409–7.223)*
Insufficient sex education	1.032	.394	2.806 (1.296–6.077)*

**Violent recidivism** *Full model: chi-square 19.912, df 3, N = 125, Nagelkerke R^2 ^0.209*			

Group offending	1.419	.461	4.135 (1.676–10.199)*
Insufficient sex education	1.173	.471	3.233 (1.284–8.137)*
Lack of empathy	.847	.433	2.333 (0.999–5.449)*

**Multiple sex offending** *Full model: chi-square 33.212, df 2, N = 118, Nagelkerke R^2 ^0.334*			

Problematic psychosexual development	1.854	.445	6.387 (2.667–15.291)*
Impulsive offense	1.122	.446	3.072 (1.283–7.355)*

*Note*. * p < 0.05

Based on their criminal history (as determined through official registration systems) two groups could be distinguished: boys who sexually offended only once and boys who sexually offended more than once (boys who committed more than one sexual offense before or around the time of the index offense: multiple sex offenders). Regression analysis showed that problematic psychosexual development and an impulsive offense distinguished multiple sex offenders from once only sex offenders (see table 6).

## Discussion

This study aimed to describe offense related characteristics and the psychosexual development of (subgroups of) juvenile sex offenders and the predictive validity of these characteristics for persistent (sexual) offensive behavior.

Assessment of (subgroups of) juvenile sex offenders by means of the BARO and GAIJSO revealed high to very high concern on several domains. The greatest need for extensive diagnostic assessment was seen in the group of child molesters. These youngsters displayed more internalizing and (psychosexual) developmental problems and their sexual offense was more alarming as compared to the other juvenile sex offender subgroups.

Although a substantial proportion of the subjects committed more than one sexual offense before or around the time of the index offense, almost no sexual recidivism was found afterwards. However, a substantial number of the juvenile sex offenders showed non-sexual and violent recidivism. Group offenders displayed more non-sexual and violent offensive behavior before and after the index offense compared to solo offenders and child molesters. Non-sexual recidivism was predicted by group offending, taking no responsibility and insufficient sex education, and was negatively associated with having a male victim. Violent reoffending was predicted by group offending, insufficient sex education and lack of empathy. Insufficient sex education, as a predictor of non-sexual (including violent) recidivism, is probably an expression of a wider concept of insufficient education at school and/or at home. Being a multiple sex offender was predicted by problematic psychosexual development and an impulsive offense. Although it can not be concluded from this finding that these items predict sexual recidivism, further exploration of psychosexual development and impulsivity in relation to sexual reoffending, measured prospectively, is warranted.

Child molesters displayed more alarming results as compared to the other juvenile sex offender subgroups. Interestingly, compared to solo rapists and group offenders, child molesters showed higher rates of internalizing problems, a more alarming psychosexual development, and had more frequently a history of sexual abuse. For treatment purposes, it may be relevant to find out whether these conditions were present before or as a consequence of the offense. If present before, feelings of depression or anxiety may well have increased vulnerability towards offending. Child molesters reported to be frequently sexually abused themselves, which may have led to these internalizing problems or to Posttraumatic Stress Disorder (PTSD). Hendriks and Bijleveld [25] did not find a difference between child abusers versus peer abusers with respect to a history of sexual abuse. This discrepancy might be explained by the different subgroups used in the current study in which peer abusers were further subdivided into group peer abusers versus solo peer abusers. Child maltreatment (physical and sexual victimization) is related to PTSD and delinquency. PTSD positive delinquents are found to be most troubled in terms of impulse control and control of aggression [26]. Further research on child molesters should include diagnostic assessment of PTSD, impulse control and conduct disorders. If feelings of depression or anxiety have arised as a result of the offense, these conditions may well be a congruent reaction to a shameful situation. Given the aversion from society towards juvenile sex offenders, and child molesters in particular, this was expected to some extent. In that case, it should be seen whether the conditions are temporary and in little need of treatment.

According to Worling and Långström [27], adolescents who commit sexual offenses are more likely to be apprehended for non-sexual reoffenses than for sexual reoffenses. In line with this finding the group of juvenile sex offenders studied displayed mostly non-sexual (non-sexual 55.6% and violent 32.1%) reoffenses. Van Wijk et al. [28] found in their Dutch sample of juvenile sex offenders mostly non-sexual reoffenses as well. In agreement with the literature on adult sex offenders, Worling and Långström found a significant linear relationship between length of follow up period and sexual assault recidivism as well as for any criminal recidivism. The low rate of sexual recidivism in this sample might be due to the relatively short follow up period (mean follow up period: 36 months SD 18 months) and therefore a longer follow up period seems desirable.

With respect to the criminal careers of juvenile sex offenders, Becker & Kaplan [29] distinguished, on the basis of their clinical experience, three pathways. After their first sex offense, juvenile sex offenders can proceed onto the dead end, delinquent or deviant sexual pathway. Yet it is still unclear onto which pathway the subjects are proceeding. Some boys seem to proceed onto the dead end pathway, and others might be on the general delinquent pathways. One third of the subjects had already perpetrated multiple sex offenses at the time of the index offense, which may indicate that the boys were already on the sexual deviant or the general delinquent pathway at the time of the index offense. A longer follow up period is needed to interpret these findings adequately.

Investigation of juvenile sex offenders is important in order to recognize causal relationships and (treatable) problems of juvenile sex offenders. Using the GAIJSO, specific sexual offense related characteristics can be investigated. The information on offender type (child molester versus rapist) and way of offending (solo versus in groups) alerts the professional to pay attention to specific problems associated with these types of offenders. The information assessed by means of the GAIJSO on problematic psychosexual development points the way to further diagnostic assessment and treatment, and prevention programs can directly address the problems observed. Assessment with the GAIJSO gives insight into the specific problems of juvenile sex offenders which remain underexposed after general assessment with the BARO or other instruments. Its supplementary value lies in the fact that the GAIJSO can be used to distill relevant information in a standardized way from police records. Moreover, it collects valuable information on the sexual offense and psychosexual development of a youngster in a semi-structured way. The information obtained is of added value to the report of the child welfare counselors. It contributes to a well considered advice to the judicial authorities and is important in initiating immediate and appropriate care.

This study revealed numerous problems in juvenile sex offenders. Assessment using the GAIJSO gives insight into these specific problems of juvenile sex offenders, which remain underexposed after general assessment by means of the BARO or other instruments. Several differences were found between subgroups of juvenile sex offenders, with the most alarming results in the group of child molesters. These findings should be further investigated in an international study with a follow up interval of at least five years.

### Limitations

Findings of this study should be interpreted in light of some limitations. First of all, because of the relatively low prevalence of sex offending, it is difficult to assess large groups of sex offenders for research goals. For that reason, specific subgroups such as child molesters, were small in the present study.

A second limitation of this study is that there is no control group available of youngsters who (allegedly) did not commit a sex offense. Although, for instance, sixty percent of the young offenders showed sexual preoccupation and approximately sixteen percent had deviant sexual fantasies, it is unknown if this might be the *base rate *for every youngster, i.e., being age appropriate.

Third, at follow up, recidivism was determined only from official registration systems. Offenses unknown to police, the so-called dark number, are unknown. However, reliability of self-report questionnaires may be questioned as well, as recidivists should not be expected to report unregistered offenses willingly.

Fourth, the follow up period was relatively short, which might explain the low rate of sexual recidivism. In order to investigate the predictive validity for sexual reoffending, a longer follow up period seems desirable.

Fifth, the psychometric properties of the GAIJSO, as inter-rater reliability or test-retest reliability, have not been established yet, however, the current study does show the expected characteristics of juvenile sex offenders.

## Competing interests

The authors declare that they have no competing interests.

## Authors' contributions

All listed authors have key responsibility for the material in the article. All authors (L'tH, TD, LJ, AvW and RB) have made substantial contributions to the conception and design, acquisition of data, or analysis and interpretation of data. All authors have been involved in drafting the manuscript or revising it critically for important intellectual content and have given final approval of the version to be published.
